# Identification of a decedent in a 103-year-old homicide case using forensic anthropology and genetic genealogy

**DOI:** 10.1080/20961790.2022.2034717

**Published:** 2022-03-11

**Authors:** Amy R. Michael, Samantha H. Blatt, Mariyam Isa, Anthony Redgrave, Douglas H. Ubelaker

**Affiliations:** a Department of Anthropology, University of New Hampshire, Durham, NC, USA; b Department of Anthropology, Idaho State University, Pocatello, ID, USA; c Department of Anthropology, Texas Tech University, Lubbock, TX, USA; d Redgrave Research Forensic Services, Orange, CA, USA; e Department of Anthropology, National Museum of Natural History, Smithsonian Institution, Washington, DC, USA

**Keywords:** Forensic sciences; forensic anthropology, forensic genetic genealogy, human identification, cold case, investigative genetic genealogy, postmortem interval, skeletal trauma

## Abstract

Anthropologists are often the custodians of long-term unidentified human remains though their positions as curators of university or museum skeletal collections. Various factors decrease the solvability of these legacy cases including the passage of time, the loss of provenience for specific cases, and lack of documentation or case records. While anthropologists can contribute important information toward identification, it is often necessary to explore novel and cross-disciplinary strategies to resolve difficult cold cases.

In long cold cases, the postmortem interval, in particular, may be difficult to estimate leading to further challenges in achieving identification. Modern advances in radiocarbon bomb pulse dating, isotope analysis, and actualistic studies have contributed to positive identification of unidentified human remains in some legacy cases, but may not be available to all forensic practitioners and law enforcement from resource-poor agencies. Pooling resources, as well as collaborating with professionals outside of forensic anthropology, is a useful strategy to pursue when anthropological methods are exhausted.The case study presented here demonstrates a collaborative approach between forensic anthropologists, forensic genetic genealogists, and law enforcement in a century-old homicide. The dismembered and mummified parts of a male body were recovered in a remote cave in 1979 and again in 1991. Despite forensic anthropologists creating and updating the biological profile over the decades from recovery to present, no identification was made until the application of forensic genetic genealogy (FGG) to the case in 2019. New interpretations of bone microstructure and trauma analysis are presented for the case, alongside the historical documentation and “proof of life” evidence used by the genealogy team. A review of the FGG methods underscores the challenges in this case (e.g. significant endogamy, multiple aliases used by the victim) and the steps taken toward resolution. Ultimately, a combined anthropology and genealogy approach resulted in a confirmed identity for a man who was murdered in 1916.

**Key points**
- Forensic scientists should leverage a collaborative, interdisciplinary approach toward human identification.
- When combined with forensic anthropology methods, forensic genetic genealogy is a valuable tool linking biological and cultural-historical aspects of identity.
- Forensic anthropologists should review challenging cases in their labs as new methods are introduced and new resources become available.

## Introduction

Recent identifications of decedents and perpetrators in long-cold cases have demonstrated the application of forensic genetic genealogy (FGG), alternatively referred to as investigative genetic genealogy, in case resolution [1–6]. Used in cases involving unidentified human remains and unknown perpetrator profiles, FGG methods have contributed to solving approximately 100 cases, overwhelmingly in the US, as of 2020 [7], even resulting in identification in a case from the mid-19th century. While traditional genealogy utilises public records and family histories, FGG goes further by relying on data gathered from direct-to-consumer (DTC) ancestry kits (e.g. AncestryDNA, 23andMe, FamilyTreeDNA, MyHeritage), publicly accessible DNA databases (e.g. GEDMatch, FamilyTreeDNA), and careful interpretation of individual ancestry results. DTC ancestry kits allow for connections between extended and/or unknown family members who have uploaded their personal profiles to public websites [1]. Genealogists develop family trees, in association with data on age, sex, and geographic location, to target potential matches and interpret links between the trees.

By combining genealogical research and archival documents with DNA data, researchers can test hypotheses about biological relationships between known and unidentified individuals. This approach is not foolproof or without complications, as statistical results need to be interpreted and supported with evidence. FGG methods should not be misunderstood as a replacement for thorough investigations from law enforcement or analyses from anthropologists, but rather as a complementary approach in difficult cold cases. In many cases, genealogical input can significantly direct an investigation by narrowing the number of possible victims (or perpetrators) to a handful of individuals if not a single individual [1]. Once the victim or suspect pool has been winnowed by “shrinking the haystack” [2], the work of anthropologists and law enforcement can aid in identification and case resolution.

The development of FGG non-profits (e.g. DNA Doe Project; Trans Doe Task Force) and increased reliance on FGG methods by law enforcement is outpacing the discussion of methodology in the academic branches of the forensic sciences. Most academic publications have focused on the bioethics and legal ramifications of using FGG to identify victims and perpetrators [8–12], without much emphasis on the collaborative nature of FGG work [13]. Forensic anthropologists are uniquely suited to collaborate with forensic genetic genealogists on cold cases as anthropologists often curate unidentified human remains in their labs and/or are the consultants called by the medicolegal authority when skeletonised and decomposed remains are recovered [14].

Here, we present an example of a successful collaboration in the investigation of a challenging cold case that were resolved through the prolonged and collaborative efforts of law enforcement, anthropologists, DNA analysts, and genealogists. The intersections of these fields, as well as the data produced from each, were leveraged to narrow potential matches and resolve the identity of a homicide victim in a historic cold case. Positive identification was initially hindered by the absence of key elements (the skull) and a challenging postmortem interval (PMI) due to the inability to predict decomposition rates in a cave environment [15]. As forensic anthropologists explore novel and cross-disciplinary strategies to resolve difficult cold cases, we emphasise that one potential avenue toward resolution is the establishment of a close working relationship with FGG. Though forensic anthropologists can often effectively estimate the biological profile, identify and describe trauma, and provide critical context clues to law enforcement, they should also consult other forensic subject matter experts when anthropological expertise has been exhausted to reconstruct a holistic biocultural profile of a decedent.

### Background of the case

In late August 1979, a family searching for artifacts in a lava tube cave near the small eastern Idaho town of Dubois reported finding clothing in a burlap sack shallowly interred in soft loamy soil approximately 200 feet/71 m from the western cave entrance. The family notified law enforcement and the Clark County Sheriff’s Office responded and recovered a clothed mummified and headless human torso. The associated clothing was described as a pink or red shirt with blue pinstripes, maroon wool knit sweater, and black wool pants with suspenders. A visual search, use of metal detectors, and 2.5 feet/0.76 m excavation beneath the torso to bedrock yielded no other remains at the time. The sheriff recorded the temperature (44°F/6.67°C) and humidity (66%) of the cave and loamy sand the night of the recovery. The cave remains at this temperature year-round according to local law enforcement.

The remains were well-preserved with a noticeable odour, leading the coroner to speculate that the body had been in the cave for no more than 10 years. The sheriff disagreed on the basis of associated clothing which was machine-stitched but outdated, leading him to conclude that the remains were likely those of a “long ago gambler” [16]. At the time, local law enforcement had no leads for identification and the remains were transferred to the Federal Bureau of Investigation (FBI) for examination.

In September 1979, the torso was examined by Smithsonian anthropologist Dr. Douglas Ubelaker who confirmed the victim was male based on soft tissue preservation. Ubelaker estimated that the decedent was at least 40 years old at the time of death based on the pubic bone morphology. He also cut a thin section from the left femoral midshaft to use in histological age-at-death estimation [17,18]. Reddish-brown pubic hair remained on the body which was consistent with a “probable white racial affiliation” [19]. According to both the Clark County coroner and Ubelaker, the head was removed approximately one inch/2.5 cm from the shoulders at the neck, the arms were severed at the mid-shafts, and the legs were removed below the hip. Ubelaker also noted that no “unusual perforations” were found on the torso, as dismemberment trauma was restricted to the appendicular elements and the decapitation.

In partial agreement with the coroner, Ubelaker estimated that the victim had been deceased for approximately 6 months to 5 years based on the overall preservation of the mummified, leathery skin and persistent odour of the remains. However, he acknowledged that the possibility existed for a greater PMI [15] likely due to the unknown taphonomic effects of the cave. The mummified hands were removed and sent to the FBI for latent fingerprint analysis. No matches were ever made to the decedent’s fingerprints. With identification stalled, the torso was returned to Idaho and buried in a local cemetery.

In late March 1991, another family digging for artifacts in the same lava tube discovered an exposed mummified human hand. Law enforcement responded and recovered four clothed limbs inside a burlap sack [15]. A systematic search and excavation of the cave was conducted in April 1991 with the Clark County Sheriff’s Office and anthropologists from Idaho State University (ISU). Anthropologists probed the soil across the width (90 feet/27.4 m) and length of the cave (0.5 mile/805 m) to search for the skull and any other evidence. Intersecting trenches were dug between the areas where remains were recovered in 1979 and 1991, but no additional remains or other evidence were located.

The buried torso recovered in 1979 was then exhumed and confirmed as a match to the severed limbs. In 1997, the remains were transferred to the ISU Anthropology Department for secure storage and curation. Another search of the cave by anthropologists, law enforcement, and a private K9 cadaver dog handler was mounted in February 2015, but again yielded no skull or further evidence. A bone sample submitted for DNA in 2017 returned no matches from the Combined DNA Index System (CODIS).

## Materials

**Figure 1.**
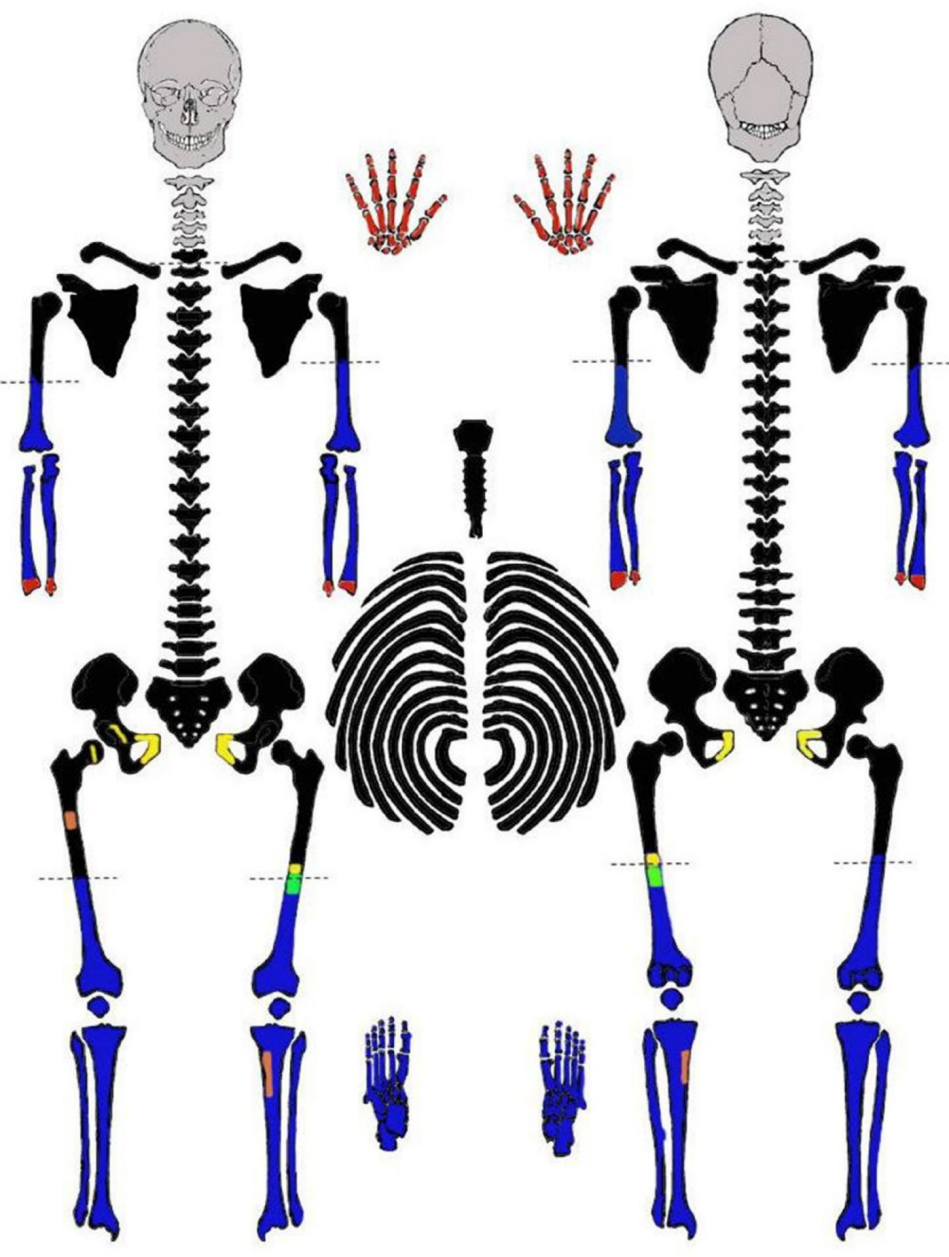
Skeletal inventory of Joseph Henry Loveless presented as a case timeline. Gray elements indicate those never recovered. Black elements were recovered in 1979. Blue elements were recovered in 1991. Red areas were cut and sampled by Federal Bureau of Investigation (FBI) 1979 and not present for current analysis. Yellow areas indicate cuts made in 1979 by the Smithsonian. The green area indicates the histological section made by Idaho State University (ISU) 2019. The orange areas indicate the bone window made on the right femur in 2015 for DNA sampling (sent in 2017), and the 2019 DNA sampling of the left tibia. Dotted lines indicate perimortem dismemberment locations.

The skeletal remains were in good condition with minimal taphonomic damage. All skeletal elements were ultimately recovered except the skull (and teeth) and cervical vertebrae 1–5, representing one individual. The hands, including the wrists, and distal ends of both radii and ulnae were removed in 1979 by the FBI attempting to get fingerprints from the mummified tissue and were never reassociated with the rest of the body in Idaho. The location of these elements is unknown. Similarly, the pubic bones were removed when mummified tissue was still covering the pelvis, leaving cuts on the right femoral head and right lunar surface of the acetabulum. These bones were also not reassociated with the body. In 1979, a section of the left femoral midshaft was removed for histological analysis and the slide is retained at the Smithsonian. Beyond these elements, the remainder of the skeleton was complete and all features were largely intact due to the presence of mummified tissue covering the remains and low animal/insect activity after death (Figure 1).

In 2015, the mummified tissue from the remainder of the body was removed with the use of dermestid beetles. A window of bone from the right femur was removed and sent to University of North Texas in 2017 for entry into the National Missing and Unidentified Persons System (NamUs). In March 2019, anthropologists from ISU and the University of New Hampshire (UNH) worked together with the Clark County Sheriff’s Office to submit the case to the non-profit FGG group, the DNA Doe Project (DDP). This volunteer-run organisation uses FGG methods to resolve cases that have reached investigative dead ends. After funds were raised through crowd-sourcing platforms and DDP social media posts, three bones (left tibia, left fibula, and left first metatarsal) were submitted for DNA extraction. Finally, a second thin section was cut from the left femur in 2019 for additional histological analysis.

## Methods

### Biological profile

Prior to FGG work (described below), the skeletonised remains were reanalysed in 2019. As with all metrics analyses for this case, ancestry was estimated from post-cranial metrics using Fordisc software Ver. 3.1.314 [20] with all possible postcranial measurements entered, given preservation and trauma. Measurements were taken from the clavicle, scapula, humerus, ulna, radius, sacrum, innominate, femur, tibia, fibula, os coxa, and calcaneus. Data were evaluated using all modern groups in the Fordisc databank from both sexes with Forward Wilks stepwise selection with default parameters.

Post-cranial measurements were also used for sex estimation [21,22] in Fordisc [20]. Due to dismemberment, all metrics except the maximum lengths of the radii and ulnae and maximum and bicondylar lengths of femora were used. Additionally, mummified genitalia were observable on the body after recovery in 1979. Sexually dimorphic features of the pubis such as the ventral arc, ischiopubic ramus, and subpubic concavity were not available for reanalysis as these elements were removed in 1979 and never reassociated with the body.

Stature was also estimated using Fordisc [20] from the maximum lengths of the tibia and fibula and height of the sacrum. The best indicator for stature estimation, femoral length, was compromised by the dismemberment process and thus could not be used in the estimation.

Age-at-death was initially estimated from the pubic symphyses [23,24] and a histological section [18] from the left femoral midshaft by Ubelaker in 1979. Since pubic bones were never re-associated with the body, they were not reassessed with more recent methods. However, the auricular surfaces [25] were observable, and sternal rib ends were also present to estimate age-at-death [26–28] in the reanalysis.

### Histology and bone remodeling

Permission from the Clark County Sheriff’s Office was granted to remove a thin section (0.8 mm) from the midshaft of the left femur for further histological analysis in 2019. The section was cut using a slow speed Isomet 1000 saw 39.9 mm superior to the section originally made by Ubelaker in 1979. The section was then ground and lapped to a thickness of approximately 100 μm and mounted on a slide. Imaging was undertaken using an Olympus CX43 with polarisation using ImageJ [29,30] and Fiji [30] software. Images were obtained from four regions of interest (ROI) of the cortical bone of the section of the left femur: anterior, posterior, lateral, and medial.

Osteonal area (On.Ar) was measured on 25 osteons using the Fiji polygonal trace area function [30]. The criteria for measurement were that the maximum diameter was no more than twice the minimum diameter and that 100% of the osteon perimeter had to be visible in the ROI. Rate of bone remodeling is evidenced in secondary osteons and osteon population density (OPD) representing remodeling events and accrued bone density. When controlling for age-at-death, OPD variation may indicate differences in bone structure in response to mechanical stress, dietary or health status, and general lifestyle [31–34]. The number of intact and fragmentary osteons were counted within each ROI at a magnification of 10× were identified by the presence of intact cement lines and a complete Haversian canal. Fragmentary secondary osteons were identified as those without complete cement lines, overlapped by other osteons, or underlying other osteons, but with at least 10% of the Haversian canal observable. All counted osteons had their Haversian canals and cement outlines within the border of the ROI. The OPD was calculated by dividing the summed counts of intact and fragmentary osteons by the area of ROI (2.273 mm^2^) [32] and compared to other studies.

### Trauma analysis

While Ubelaker noted the dismemberment and decapitation in his 1979 case report, more detailed descriptions and analyses were not originally provided. As part of the current skeletal trauma analysis, high resolution gross photographs were taken of each element exhibiting sharp force defects, as well as from anterior, posterior, lateral and medial views of the cut surfaces. Because the original bone specimens could not be transported for analysis, one author (SHB) made impressions of the sharp force defects using Coltene-Whaledent Affinis Regular Body impression material. Photographs and impressions were sent for further analysis to another author (MI), who cast the impressions using FastCast urethane casting resin. Molds and casts of the sharp force defects were examined and photographed macroscopically as well as microscopically using a Dino-Lite 5MP Edge AM7115MZT digital microscope (AnMo Electronics Corp., Taiwan, China).

Various systems for categorising dismemberment cases have been proposed, for example based on the location of cut marks [35] or the perpetrator’s motive [36]. Rainwater [37] instead proposes three modes of dismemberment based on the pattern of trauma and class of tool employed. This system was used to evaluate the trauma in the current case, as it is informative about the method of dismemberment without overstating the probative value of the physical evidence by assigning motive. Alterations were described and, where possible, some blade class characteristics were evaluated following Symes et al. [38] and others.

### Forensic genetic genealogy

A sample from the left tibia yielded 76.2 ng during the DNA extraction process and was submitted for whole genome sequencing and bioinformatics to produce an uploadable data file compatible for comparison on GEDmatch (www.gedmatch.com) and FamilyTreeDNA (FTDNA, www.familytreedna.com). Sites like GEDmatch allow individuals who have DTC results to upload their raw data and compare them to other kits in the system. FGG involved analysis of hundreds of thousands of autosomal single nucleotide polymorphisms (SNPs) to generate a DNA profile [13]. This is much more data compared to the 20 loci within short tandem repeats (STRs) used in traditional forensic genetics.

The genetic distance of nucleotides was measured in centimorgans (cM), in which 1 cM equals a 1% probability of recombination. More closely related individuals will share longer segments of DNA (> 6 cM) that are considered identical-by-descent (IBD) [1,5,39]. Through generations IBD segments become shorter with more recombination events, so lengths of IBD segments (in cM) were used to estimate the relative degree of biological relatedness. The Shared cM Project (ScP), a compilation of autosomal DNA ranges and means based on a survey of the amount of DNA shared by individuals with known relationships, is used to estimate familial relationship [13]. A conditional probability tool, What Are The Odds (WATO) from DNAPainter (www.dnapainter.com) [40] based on ScP statistics was then used to generate probability trees of the hypothetical relationship of the individual to genetic relatives based on their total shared cMs. However, since statistical ranges of relatedness can overlap with more than one type of familial relationship, document searches are used to narrow possibilities.

The anthropological analyses (refined biological profile, trauma analysis, and histological analysis) provided genealogists contextualised information to interpret potential matches. Genealogists also used newspaper databases to collate aliases and additional documentary evidence, or “proof of life” evidence, to establish timelines and personal details. These details were summarized for law enforcement before a familial “target tester” was identified to confirm the match to the decedent.

## Results

### Ancestry estimation

Assuming that this individual belongs to one of the four groups they were compared to (White Male, White Female, Black Male, Black Female) in a discriminant functions analysis using 33 of a possible 42 postcranial measurements, Fordisc [20] results indicated that this individual is closest to the White Male sample (posterior probability = 0.919; typicality probability ≤ 0.01). This finding was consistent with Ubelaker’s [19] original report indicating the decedent was likely white due to the reddish-brown hair preserved on the mummified skin.

### Sex estimation

Overall skeletal morphology was robust and consistent with male. Following Spradley and Jantz [21], the intact post-crania (sacrum, tibia, fibula) were estimated to be from a male individual. Measurements of the right and left femoral and humeral heads, and the right and left glenoid fossae of the scapulae were taken from the post-crania and also estimated to be from a male [22]. These results are consistent with Ubelaker’s 1979 [19] examination of the pubic bones and genital morphology.

### Age-at-death estimation

In 1979, Ubelaker observed rim development around the pubic symphysis [23]. When assessing age from a histological section of the left femur using Kerley and Ubelaker [18], no primary osteons were present and osteon counts (*n* = 116) and fragment counts (*n* = 39) and lamellar bone (9%) yielded an overall estimate of 42 years at death. Morphology of the auricular surfaces of the ilium [25] were consistent with phase 6 (45–49 years). Sternal rib ends [25,27,28] were examined and age was estimated to be consistent with the original report, but expanded to 35–50 years. Slight degenerative changes (marginal lipping and Schmorl’s nodes) were noted in the vertebral bodies indicating incipient arthritis. Summary age was 35–50 years.

### Stature estimation

The decedent was compared to White Male groups in Fordisc [20] from the 19th century and modern samples. The results from the tibia, fibula, and sacrum indicated the decedent was between 5′6″ (167.64 cm) and 6′2″ (187.96 cm) with a 95% prediction interval.

### Bone remodeling

Overall, the calculated OPD is higher (Table 1) than in most reported populations, including European males by the age class [32,41]. As Burr et al. [31] and Robling and Stout [42] have demonstrated, a more active lifestyle is associated with greater OPD. The anterior and lateral aspects of the femur show the highest density indicating increased mechanical loading and intracortical turnover in these areas (Table 1). This is consistent with the findings of OPD density of femoral shafts from a cadaver sample, which was increasingly distinctive with older age [42]. The anterior and lateral collagen fiber orientation (CFO) were directed longitudinally, reflecting tensile forces, while medial and posterior CFO were transverse, reflecting compressive or shear forces in the decedent, which is also in line with other studies [42]. These observations reflect typical human gait strategies [43]. However, mean On.Ar (0.0328 mm^2^) was small compared to Medieval England cemetery samples [32,42]. This could suggest that while the decedent was highly active, their bone remodeling was also impacted by insufficient nutrition, possibly related to socioeconomic status. However, other interpretations are certainly possible given the complexity of bone metabolism processes [32,42,44,45]. While isotopic data were not collected in this case, this information combined with life history context and bone histomorphology can contribute to a broad picture of a decedent’s lifestyle [46].

**Table 1.** Histological analysis of femur (2019).

ROI	Intact osteons (*n*)	Fragmentary osteons (*n*)	OPD
Anterior	9	25	14.958
Posterior	10	15	10.998
Medial	13	16	12.758
Lateral	16	26	18.477

ROI: region of interest; OPD: osteon population density.

### Trauma

Sharp force defects consistent with dismemberment were observed on five skeletal elements: the cervical vertebra 6 (C6), bilateral femora, and bilateral humeri.

**Figure 2.**
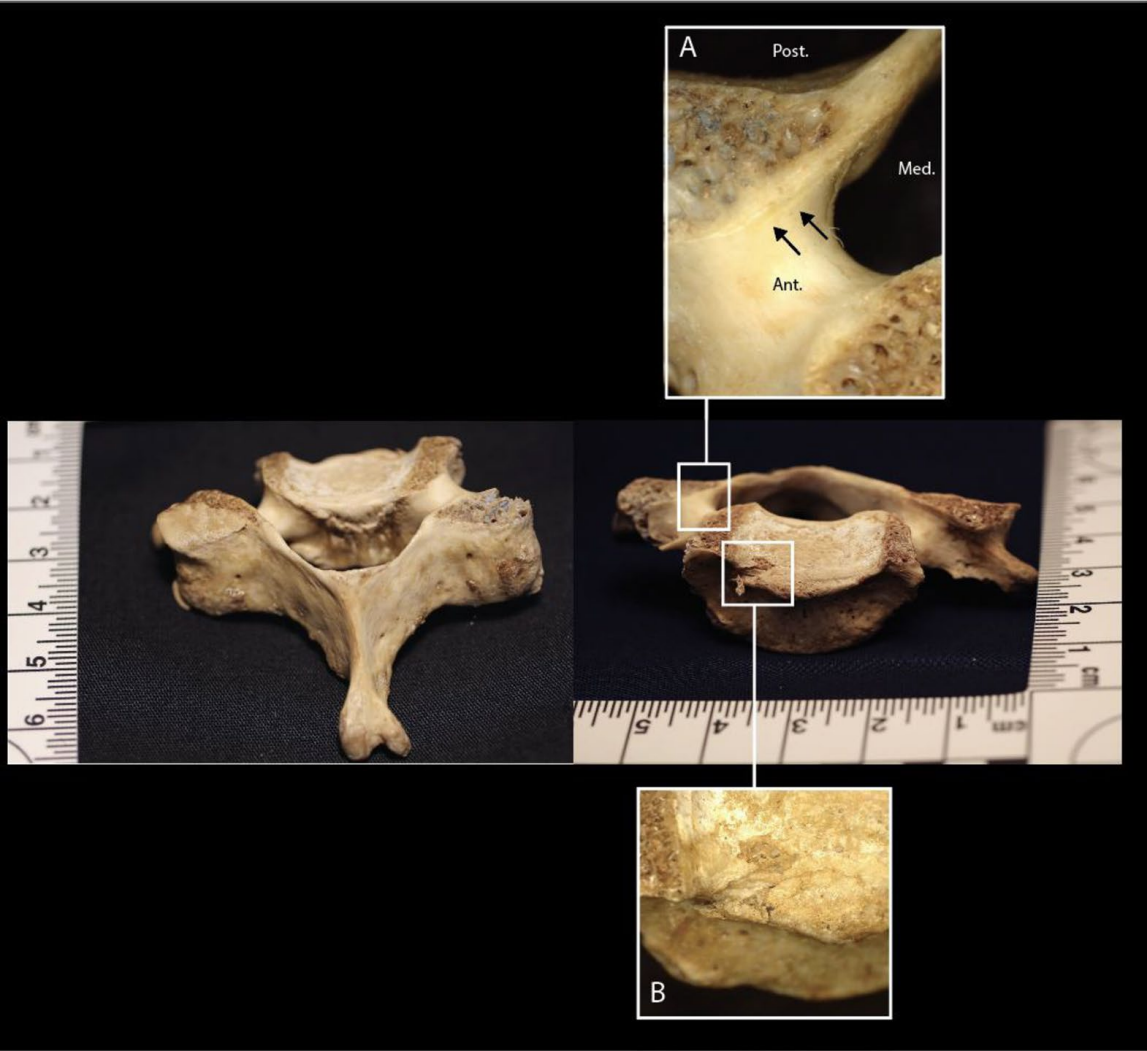
C6 vertebra, posterior–superior (image left) and anterior–superior (image right) views. Inset A: closeup of linear incised defects anterior and inferior to the defect across the right superior articular facet. Inset B: closeup of a defect on the right superior endplate.

Sharp force trauma of C6 results in the absence of the left and right superior endplate margins and portions of both superior articular facets (Figure 2). Several shallow, linear incised defects are observed anterior and inferior to the absent bone on the right superior articular facet. There is also a narrow, depressed defect on the right superior endplate, approximately 2 mm in width. Toolmark evidence was limited due to the large proportion of trabecular bone; therefore, no interpretations are presented for C6.

**Figure 3.**
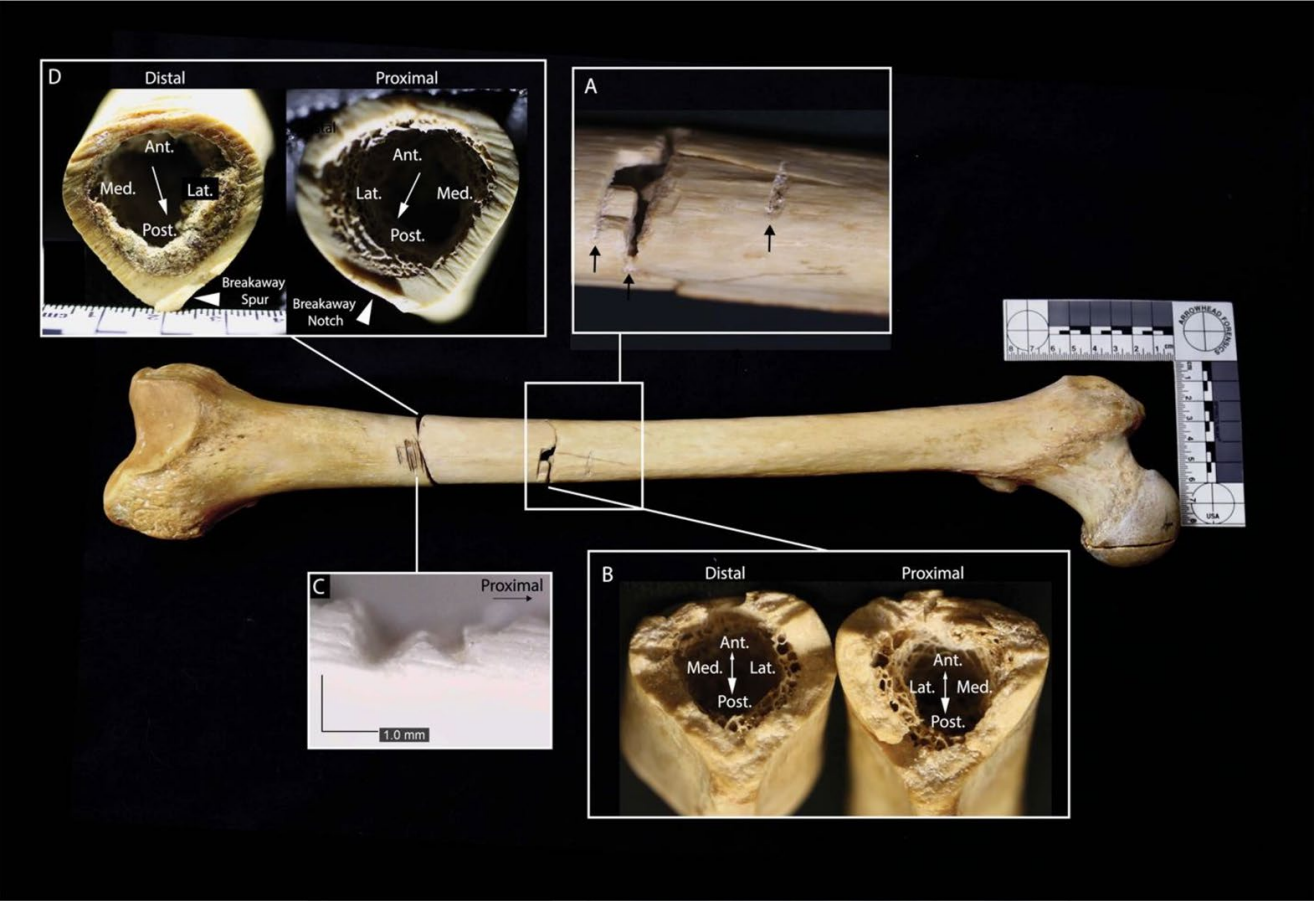
Right femur, anterior view. Inset A: closeup of defects on the anterior femoral surface; the chop marks described in the text are indicated by black arrows. Inset B: fracture surfaces of the midshaft transection; small arrowhead indicates direction of fracture propagation (from failure in tension to failure in compression) and large arrowhead indicates direction of force application (from compression to tension). Inset C: microscope image of a false start kerf on the anterior femoral surface associated with the distal shaft transection. Inset D: kerf walls of the distal shaft transection; arrows indicate direction of blade progress. Note: the femoral head cut mark was made by analysts at the Smithsonian in 1979.

The right femur is transected in two locations: the distal midshaft and the distal shaft (Figure 3). The anterior aspect of the midshaft transection exhibits a V-shaped kerf, while the surfaces of the transection exhibit primarily blunt force failure morphology (bone mirror, bone hackle, and arrest ridges) [47]. This indicates a blunt force impact by a heavy object with at least one sharp edge. The presence of a butterfly fracture with cracks branching anteriorly indicates that the bone failed in tension on the posterior surface, consistent with compression on the anterior surface [48]. The midshaft transection is also associated with two incomplete, V-shaped kerfs on the anterior aspect of the femur. The kerf wall of the more distal defect exhibits microscopic, unpatterned striations perpendicular to the kerf floor, consistent with chopping [49]. Striations were not visible macroscopically or microscopically on the kerf walls of the other midshaft defects. The trauma to the right femoral midshaft is consistent with a minimum of three chop wounds by a heavy object with a sharp edge.

The right distal femur exhibits a complete transection and several associated false starts. Kerfs are square in cross section with W-shaped floors, indicating the use of a saw rather than a tool with a beveled edge, such as a knife [38]. Kerf walls exhibit non-uniform, non-parallel striations, consistent with a hand-powered saw [38]. The position of false starts on the anterior cortical surface and a breakaway spur and notch on the posterolateral aspect of the femur indicate blade progress from approximately anteromedial to posterolateral [38].

**Figure 4.**
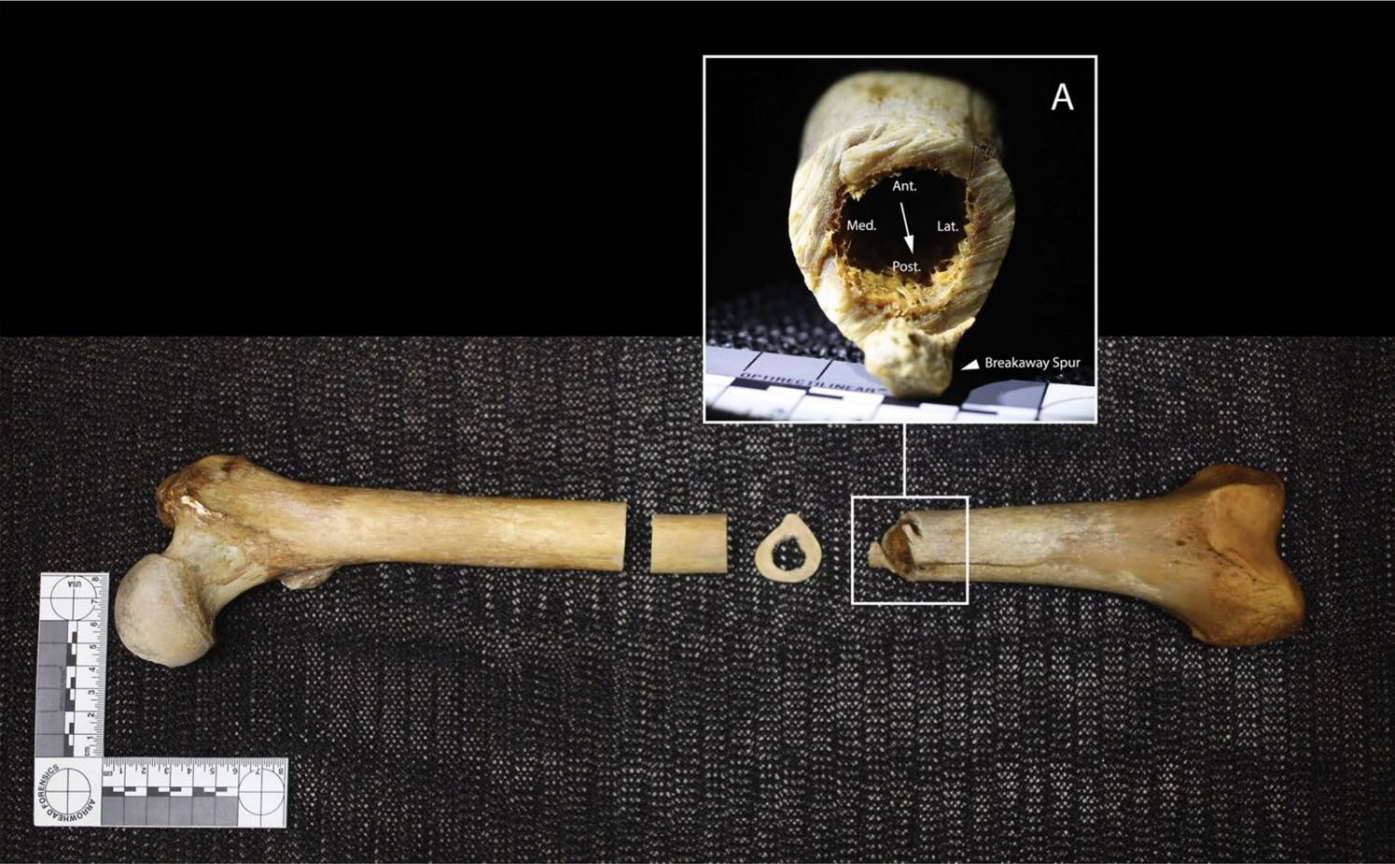
Left femur, anterior view. Inset A: distal kerf wall; arrows indicate direction of blade progress. Note: the midshaft transections are associated with sampling for histological analyses in 1979 and 2019.

The left femur exhibits a complete transection of the distal shaft (Figure 4). The position of a false start on the anterolateral aspect of the femur and a breakaway spur on the posterior aspect indicate blade progress from approximately anterior to posterior [38]. Kerfs are square in cross section and striations on the kerf walls are non-uniform and change planes throughout the cut, consistent with a hand-powered saw [38].

**Figure 5.**
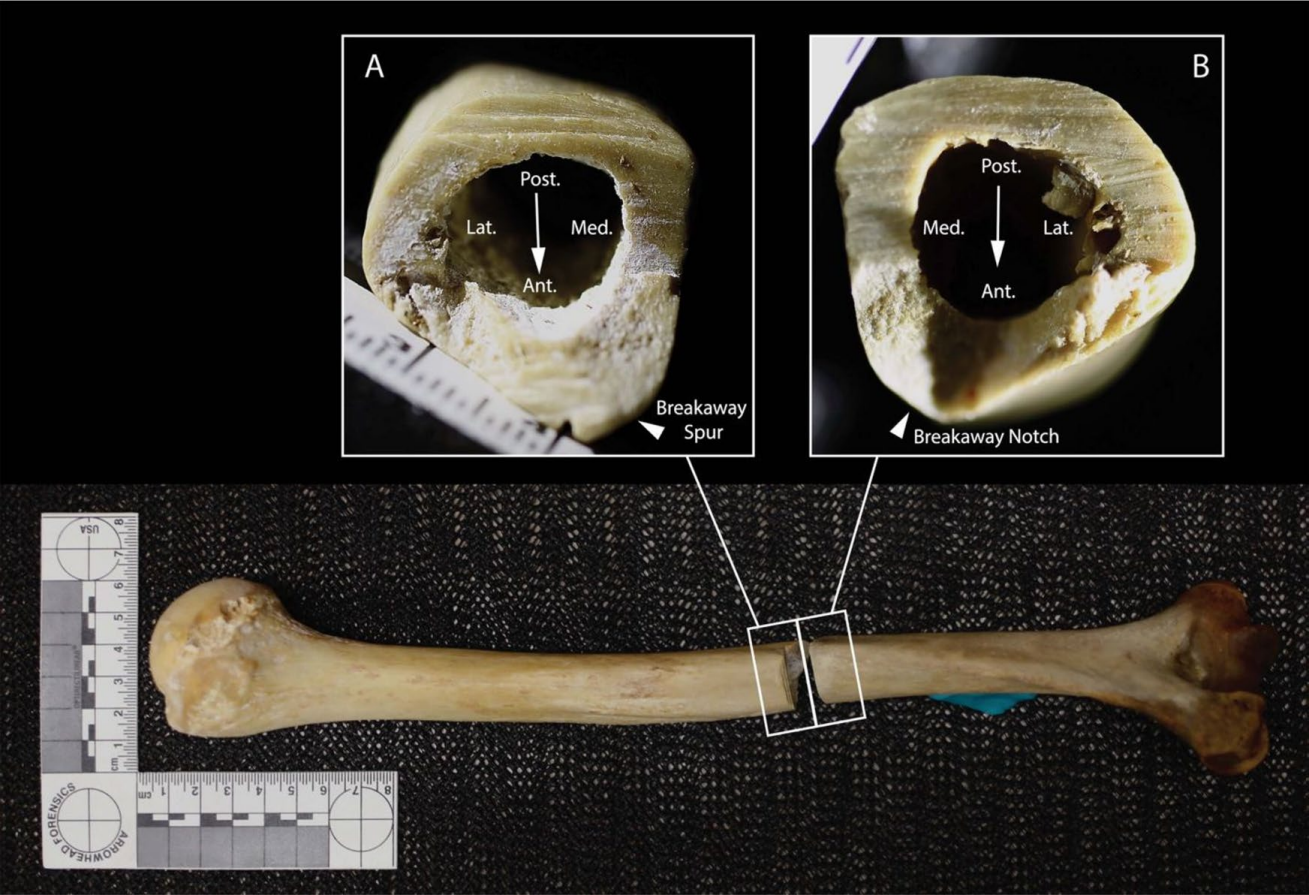
Left humerus, posterior view. Inset A: proximal kerf wall. Inset B: distal kerf wall; arrows indicate direction of blade progress.

**Figure 6.**
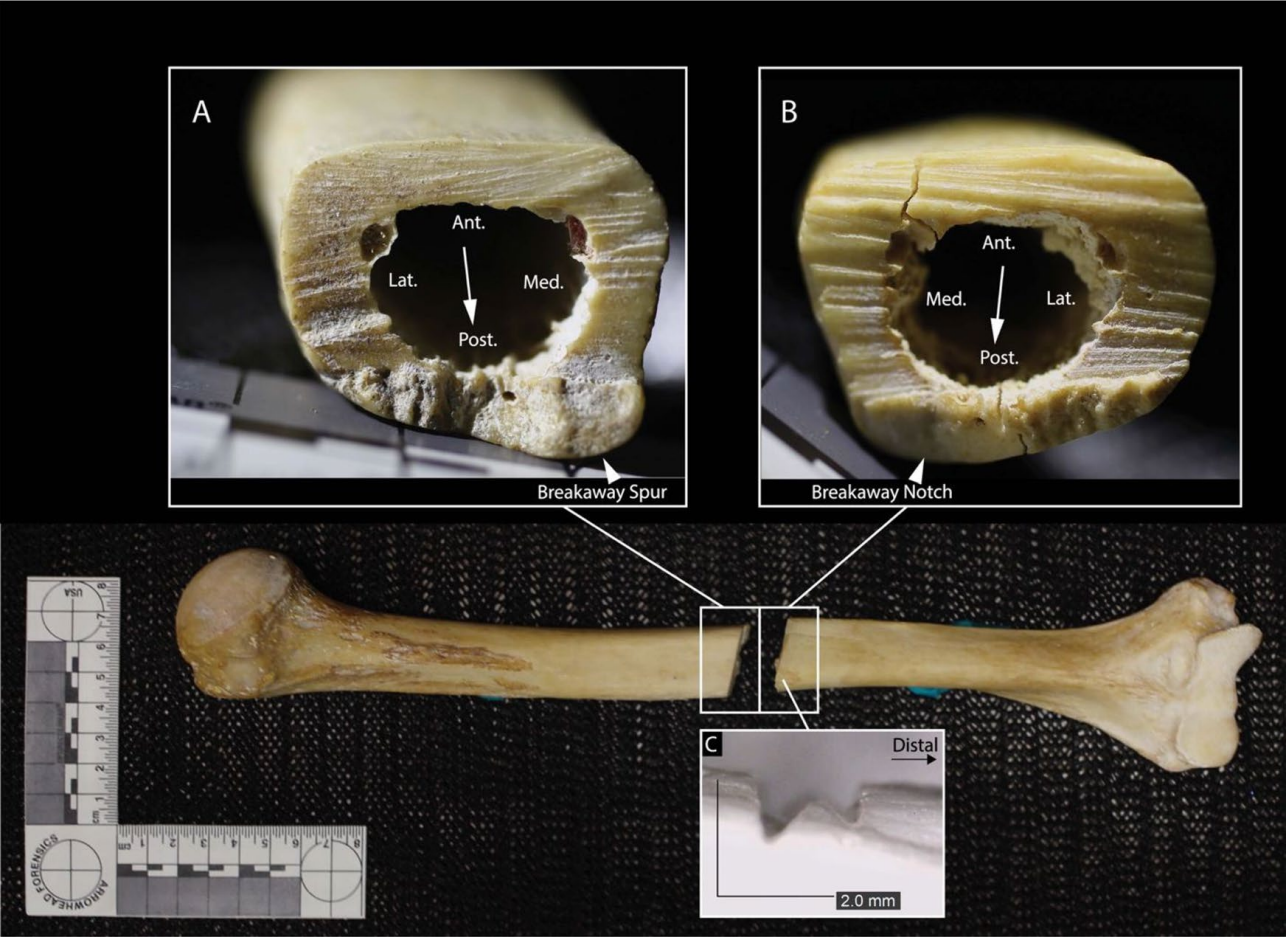
Right humerus, anterior view. Inset A: proximal kerf wall. Inset B: distal kerf wall; arrows indicate direction of blade progress. Inset C: microscope image of a false start kerf on the anterior surface of the distal section.

Both humeri are transected near the midshaft. In the left humerus, no false starts are observed, but the position of a breakaway spur and notch on the anterior aspect of the bone indicates blade progress from approximately posterior to anterior (Figure 5) [38]. In the right humerus, the positions of a false start on the anterior cortical surface and a breakaway spur and notch on the posterior aspect of the bone indicate blade progress from approximately anterior to posterior (Figure 6) [38]. The false start kerf is square and exhibits a W-shaped floor consistent with a saw [38]. The kerf walls of both humeri exhibit non-uniform striations suggestive of manual sawing [38].

The sharp force defects in this case are consistent with a minimum of two modes of dismemberment: chopping and sawing [37]. At least two tools were used to dismember the body: a heavy object with at least one sharp edge was used to chop the right femoral midshaft, while a blade was used to saw the right distal femur, left femur, and humeri. Residual characteristics including square kerfs with W-shaped floors and the presence of non-uniform, non-parallel striations across the sawed elements are consistent with the use of a hand-powered saw [38]. Minimum kerf widths of saw cuts across elements are within a similar range (1.4 mm to 1.7 mm).

Studies of modern forensic cases indicate that dismemberments are relatively rare and are almost always associated with homicide [36,50–53]. Dismemberment is often used to conceal the victim’s identity or to make the transport and disposal of the remains more efficient or discreet [50]. The recovery of elements from different, concealed locations within the cave supports the hypothesis that the dismemberment in this case represents a perpetrator or perpetrators’ efforts to avoid detection while disposing of the body.

There is no way to know for certain which tools were used to dismember the body nor what motivated the dismemberment. This information is unknowable considering that there are unlikely any surviving witnesses. Interpretations and hypotheses are presented here to help contextualise the trauma in this historic case and do not represent medicolegal determinations. Regardless of the perpetrator or perpetrators’ motives, the dismemberment and dispersal of remains slowed and complicated the recovery efforts and identification process in this case. The limbs were not recovered until 12 years after the discovery of the torso and the skull has never been recovered, precluding the use of some biological profile methods and potentially obscuring additional clues that would have shed additional light on the decedent’s identity and circumstances of death.

### Forensic genetic genealogy and archival research

Working backward from the date of discovery and using the skeletal age estimation, a targeted range of birth years from 1924 to 1944 was established. After consultations with the anthropologists about the uncertainty of the PMI, the birth year range was amended to 1914–1944. Based on the analysis of the clothing from photographs, it was hypothesized that the clothing could have been manufactured as early as the 1910s as they were machined textiles with circular knitting [54].

Using GEDMatch, the decedent’s genetic admixture was determined to be predominantly of Northern European descent (consistent with the anthropologists’ estimation). The genetic pedigree was reconstructed in a collaborative family tree along with the pedigrees of other related DNA matches, many of them estimated to be no more distantly related to the individual than third cousins. Clusters of related matches were found to share common ancestors. A group of DNA matches, referred to as “Match 3” (165 cM) and “Match 4” (170 cM), descended from the couple Samuel Scriggins (1806–1879) and Ellen Prentiss (1808–1872). Another group of DNA matches, “Match 1” (185.7 cM) and “Match 2” (207 cM), descended from John Loveless (1807–1880) and Rachel Mahala Anderson (1805–1891) and were consistent (51.9% and 42.64% probability, respectively) with first cousins 3 times removed. Historical research revealed that Loveless and Anderson were early members of the Church of Jesus Christ of Latter-day Saints, and settled in Utah Territory with the early pioneers of the church in 1851. John Loveless had three additional wives, but probabilities using WATO [40] determined that the descendants of Loveless and Anderson were the closest relatives with the highest shared cM (207) with the decedent.

Several of Loveless and Anderson’s sons also had multiple wives, as was common practice at the time when church members practiced polygamy. Some of these children had as many as 14 children of their own, most of whom stayed in Utah and Idaho. The combined factors of large groups of half-siblings in a geographically isolated religious community led to a number of unions between individuals as close as half-second cousins, sharing a common great grandfather. Many of the decedent’s DNA matches were related to each other *via* more than one common ancestor. These results indicated that the decedent was also likely to relate to his DNA matches in more than one way.

Sixteen DNA matches were found that descended from John Loveless and Rachel Anderson. As these DNA matches were added to the probability tree, the probability ratio of the more distant generations from Loveless and Anderson dropped to zero; it was clear that the only possible link between the decedent and Loveless/Anderson was a grandson relationship [39]. Given that John Loveless’ youngest child was born in 1848 and estimating his youngest child might have had children into their forties, it was estimated that the decedent was likely born before 1895.

John Loveless and Rachel Anderson had hundreds of grandsons, so while the pool of potential decedent matches had been narrowed significantly, there was still no definitive match made. Therefore, a Y DNA profile was constructed from the whole genome sequence of the individual which allowed for researchers to conduct a patrilineal surname search to narrow the potential matches. This surname search yielded a single full Y DNA match who descended from a common patrilineal ancestor with the surname Lovelace or Loveless, allowing genealogists to focus only on male descendants of Loveless and Anderson who would have retained the Loveless surname. One of Loveless and Anderson’s sons, Joseph Jackson Loveless (1831–1883) married Sarah Jane Scriggins (1839–1926), the daughter of Samuel Scriggins and Ellen Prentiss. Loveless and Scriggins had four sons: Jedediah Jackson, Joseph Henry, Raphael Grant, and John Franklin Loveless. Initially, it appeared that all four of these sons had death dates that were accounted for, indicating that none of them were missing or had unknown dates and locations of death. However, a records error had been made, listing Joseph Henry’s death the same date as his brother Jedediah Jackson. A headstone for Joseph Henry existed in the Loveless family plot in Payson City, Utah, with a birth date and no death date. An archival newspaper search then yielded articles about the axe murder of Agnes Caldwell by a man named “Walt Cairns” (also known as “Charles Smith”). This man was arrested for Agnes’ murder in 1916, but claimed that her former husband had murdered her [55]. Agnes had married twice; first to William Glenn and second to Joseph Henry Loveless. Glenn was remarried and had a new family at the time of Agnes’ death. There was no immediate record of Loveless’ whereabouts at the time.

**Figure 7.**
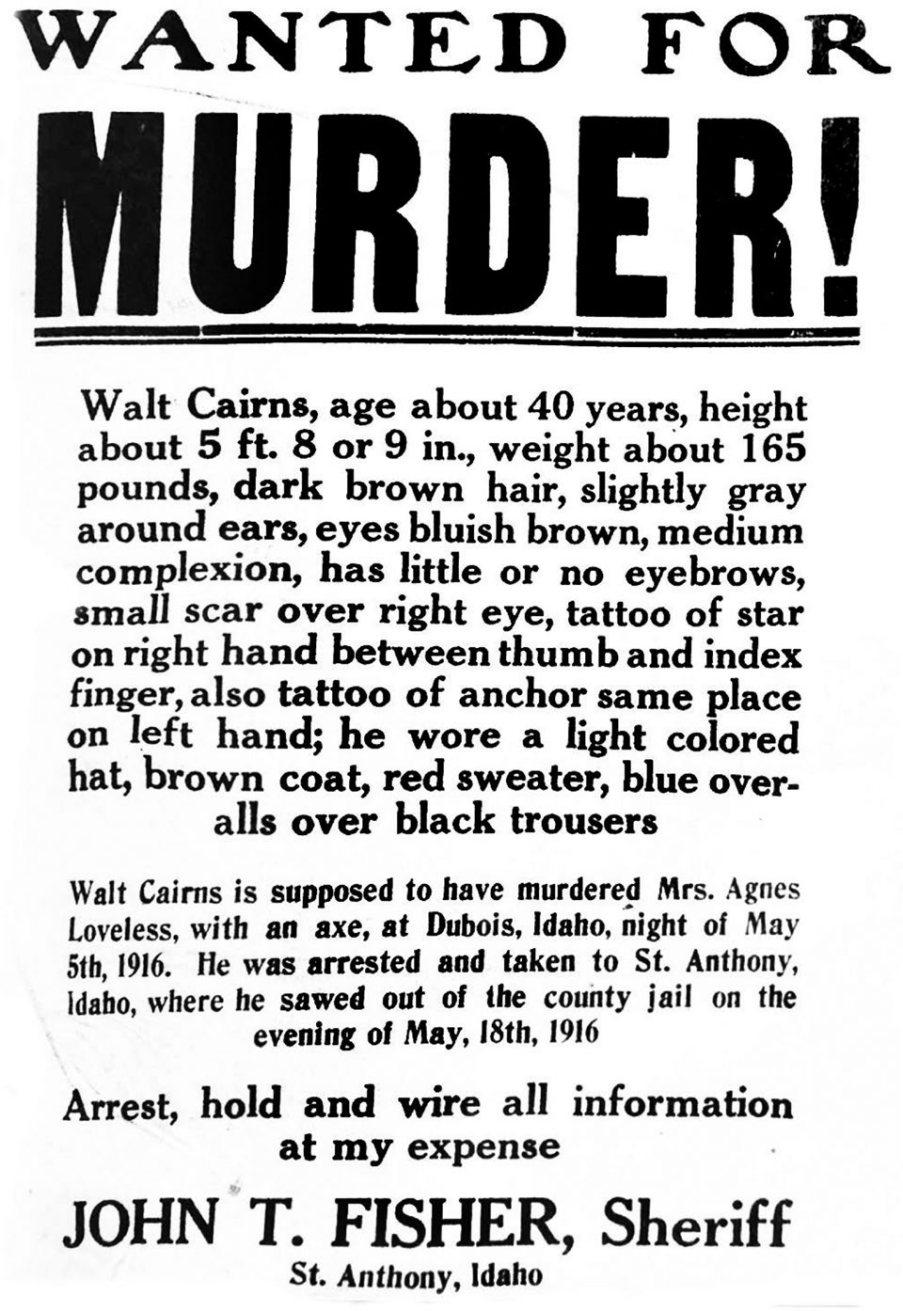
Wanted poster describing the homicide of Agnes Loveless thought to be committed by her husband, Joseph Henry Loveless (alias: Walt Cairns).

Instead of a marriage record to confirm the identity of Walt Cairns and his alleged marriage to Agnes Caldwell, a Wanted poster for Cairns was discovered in a book about historical [56] fugitive notices and wanted posters of the West. This included a physical description of his clothing at the time of his escape from jail for the murder of Agnes Loveless. He was described as wearing a “light colored hat, brown coat, red sweater and blue overalls over black trousers” (Figure 7). This description generally matched the clothing found with the decedent’s body in the cave. The genealogists deduced that since Walt Cairns was wanted for the murder of Agnes Caldwell, to whom he was supposedly married, but Agnes had been married to Joseph Henry Loveless, and the genealogical research led to the Loveless family, that Walt Cairns, Charles Smith, and Joseph Henry Loveless must all have been the same person under multiple aliases. Though he was arrested 6 days after Agnes’ murder, Loveless broke out of jail on May 18, 1916 and was never seen alive again. Beyond the matching clothing description on the Wanted poster and the clothing on the body in the cave, the circumstances of Loveless’ May 1916 escape from jail reflect his pattern of escaping previous imprisonments. One of the newspaper articles covering Agnes' funeral quoted one of their children saying, “Papa never stayed in jail very long and he’ll soon be out” [57].

**Figure 8.**
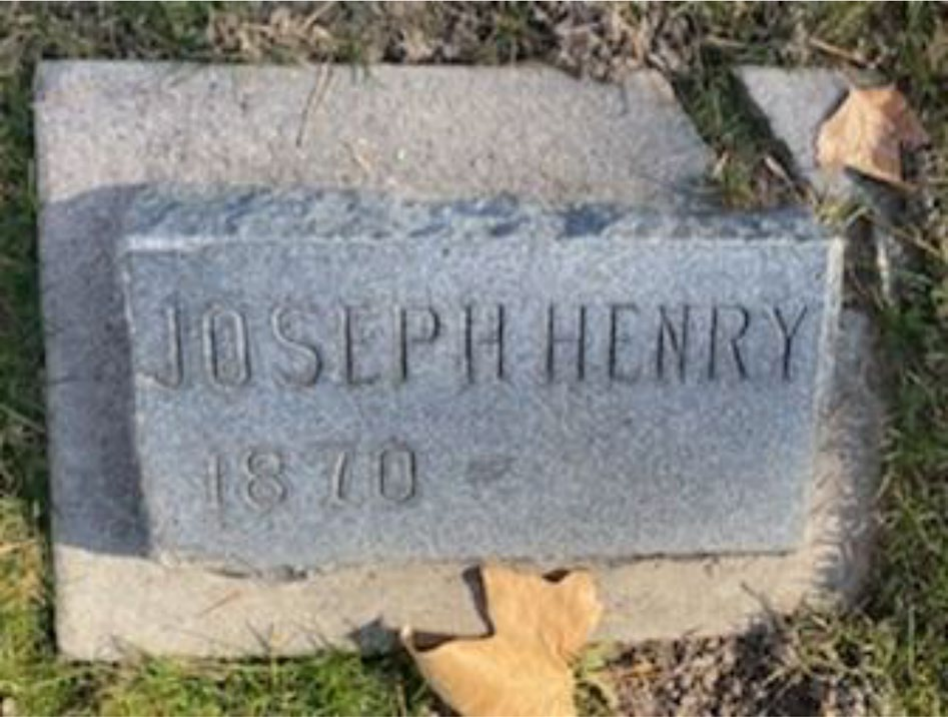
The headstone for “Joseph Henry” (born 1870) in the Loveless family plot in Payson City, UT.

Additional corroborative evidence included the discovery of the headstone with Loveless' birth date (but no death date) in a family plot in the Payson City Cemetery in Utah (Figure 8). However, no interment was ever made in the plot. The genealogists considered this further evidence that the circumstances of Loveless’ death may have been unknown to his family.

### Positive identification

The decedent was presumptively identified as Joseph Henry Loveless after 14 DDP genealogists, led by Anthony Redgrave and Lee Bingham Redgrave, worked for approximately 2 000 h over the course of 15 weeks to build the familial trees. There were over 31 730 individuals in Loveless’ genealogical tree at the time of his identification. In early November 2019, the DDP submitted the name of the candidate for identification to the Clark County Sheriff’s Office. An 87 year-old grandchild, referred to as “Match 5”, was identified as the closest living relative (1628.7 cM) and agreed to take a DTC test, which confirmed relatedness to Loveless (100% probability). The anthropological analysis (Table 2) was accurate, though the generally agreed upon PMI was off by approximately 60 years.

**Table 2.** Comparison of anthropological analysis and decedent profile after identification.

Biological profile	Estimations from skeletal remains	Joseph Henry Loveless
Age at death	35–50 years	46 years
Sex	Male	Male
Ancestry	White	White
Stature	5′6″–6′2″ (167.64–187.96 cm)	Approx. 5′8″–5′9″ (157.48–175.26 cm) (according to “Wanted” poster)
Lifestyle	High bone density which is often associated with an active lifestyle/ biomechanical loading. Small osteons possibly indicative of malnutrition or lower socioeconomic status.	Semi-transient labourer with repeat criminal behaviour

## Discussion

The cumulative challenges in the identification of Loveless could not have been surmounted without a collaborative approach, the success of which has been described elsewhere in forensic literature [13,58]. Three key issues arose during the forensic investigation: (1) reconciliation of the PMI and lack of understanding taphonomic processes in a cave setting; (2) problems in the identification of the long-term dead; and (3) application of the new method of FGG to a case held in an anthropology lab.

### Challenges in interpreting taphonomy and PMI

There are scant data on decomposition rates in cool cave settings, which led to the significant underestimation of the PMI in this case. While law enforcement recorded the ambient temperature of the cave and sand, as well as the humidity, during the original recovery in 1979, it remains difficult to determine how the shallow cave burial affected the mummification and preservation processes. Certainly, the cave environment inhibited decomposition as skin and odour was still present at the time of recovery, leading to the significantly underestimated PMI of 6 months to 5 years (actual PMI = approximately 63 years). The best sources of taphonomic and decomposition data are forensic research facilities [59,60] and none of these dedicated facilities, to our knowledge, house or mimic a cave environment. Loveless’ identification was likely prolonged by the inaccurate PMI, though all anthropologists agree that this time period was difficult to confidently determine.

### Issues in identifying long-term unidentified decedents

Another issue in long-cold cases is the loss of information, records, reports, photographs, and even body parts over time due to changing caseloads, new hires, and retiring detectives. When civilian scientists are involved, as in this case, the body parts may be moved yet again to laboratory facilities or sent *via* mail for analysis. This persistent moving process, over the course of decades, can result in the loss of crucial information and physical evidence even when no bad-faith measures are taken.

The hands were removed from the body for fingerprint analysis by the FBI in 1979, but it is unclear due to either lost or non-existent paperwork if they were ever returned to Idaho. The hands were never reassociated with the body and no matches were ever made since Loveless died long before the national database, the Integrated Automated Fingerprint Identification System, was created. For the same reason, the DNA entered into NamUs produced no matches in CODIS. In historic cold cases like this one, the inability to compare decedents to databases will always be a confounding issue.

The remote recovery site, in a very sparsely populated county, also played a role in the identification process. There were no missing persons reported in the surrounding area and the recovery of the body pre-dated systematic missing persons databases. A popular local story about a fight between two men (neither were Loveless) and arson resulting in a homicide and clandestine cave burial had been told through the years in the area. Interestingly, a local historian published a book documenting the history of Clark County and naming “Henry Lovelace” as the possible victim in the cave. Her account was based on persisting rumour and an interview with a resident alive in Dubois at the time of Agnes Loveless’ murder [61]. In long-cold cases, it may be worthwhile to consider community knowledge and lore; in this case, a historian correctly speculated the identity of the decedent, laid out some history of the decedent’s criminal history, and even reported on a potential motive of the crime. The woman who had discovered a portion of the body when she was just a child commented on social media that she was relieved to know the identity of the man as she had always dreamt about the body her family found in the cave. This is a poignant closure to Ubelaker’s chapter too [15], in which he begins by referring to the girl who discovered the body and how it may have impacted her life. In a small tight-knit community like Dubois, Idaho they embraced this case as a part of their history and the history of pioneering days of the region.

### Application of forensic genetic genealogy

The biological profile, in addition to skeletal analyses of trauma and pathology, provided a blueprint for genealogists as they built out family trees and located possible matches. Because Loveless was born into a Church of Jesus Christ of Latter-day Saints pioneer family that practiced polygamy, the genealogists were challenged by the complex endogamous relationships characterised by half-siblings. Further complicating the investigation of common ancestors and potential decedents was the broad age-at-death estimation and the uncertain PMI. The target birth and death dates were wide estimates, which necessitated hours of corroborative archival research when several candidates arose in the target range. Finally, the absence of an immediate living relative that could provide a conclusive match using one-to-one STR comparison was not possible.

Application of any method to forensic contexts must be considered carefully, especially with emerging methods like FGG. While the Loveless case is a clear endorsement of responsible application of FGG methods in human identification, other cases may be more fraught. Public opinion on law enforcement use of ancestry databases is in flux and will likely change with, and be shaped by, more layperson literacy about FGG through increased media coverage of cases involving database-related arrests or identifications. Citizens generally support law enforcement searches of genetic databases when used to identify violent perpetrators and/or missing persons [2]. Issues involving minors, informed consent, transparency and accountability in method implementation, and concerns about privacy should not be overlooked as FGG methods grow in popularity [4]. Apprehension rightfully exists about privacy, practice standards, industry regulation, and ethical guidelines as standard operating procedures have yet to be codified in this new field [1,4,12,13,62]. Issues of consent among genealogy database users remains an ethical issue to be vetted in the field [63], with trepidation about “genetic surveillance” [8]. Some states have passed legislation regulating the use of FGG, most notably in banning law enforcement from using databases to search for individuals not considered criminal suspects, while the U.S. Department of Justice has also instituted recent provisional policy guidelines for use [9,64]. As FGG methods evolve and legislation is adopted, anthropologists should stay informed about changing and culturally diverse protocols and ethical concerns for such methods for forensic, historic, and archaeological DNA. That being said, the latest revisions of GEDmatch’s privacy policy opted all profiles back into use for identification, signaling the perceived broader acceptability of this practice.

## Conclusions

While the application of some modern methods like bomb-pulse dating [65] and analysis of the clothing manufacturing and materials may have clarified the original PMI estimate and narrowed search criteria, it is clear that Joseph Henry Loveless would not have been identified without the combined methods of FGG, forensic anthropology, and detective work. Together, these efforts yielded results 40 years after the initial recovery of the remains and 103 years after the homicide. The case remains officially open per the Clark County Sheriff’s Office. The perpetrator(s) in this case are certainly no longer living, but law enforcement believes that a reasonable conclusion about the crime may be drawn as physical evidence still exists.

Anthropologists can continue to learn from this case too; a positive identification was not made in this case *because* of anthropological input, but it also could not have been made *without* anthropologists. Beyond initially advocating for FGG work and curating the remains for years, the anthropologists’ knowledge was critical to the work of the genealogists as they sifted through thousands of possible matches, while the archival documents and arrest records from law enforcement allowed for the reduction of possible matches. This case serves as a reminder for forensic anthropologists to recognize that while anthropological methods alone may be insufficient for case resolution, it is possible to advocate for the decedent by seeking other, collaborative methods for identification. Forensic anthropologists, especially those tasked with long-term curation of unidentified remains in their labs, have a clear obligation to decedents to exhaust all avenues for identification. Whenever possible, new forensic methods should be explored and critical reanalysis undertaken to achieve identification. Keeping legacy skeletal remains in an anthropology lab because they have been deemed too difficult or too old to identify is an ethically dubious approach given the rise and availability of FGG methods.

## References

[1] Greytak EM, Moore C, Armentrout SL. Genetic genealogy for cold case and active investigations. Forensic Sci Int. 2009;299:103–113. 10.1016/j.forsciint.2019.03.039 30991209

[2] Guerrini CJ, Robinson JO, Petersen D, et al. Should police have access to genetic genealogy databases? Capturing the Golden State Killer and other criminals using a controversial new forensic technique. PLoS Biol. 2018;16:e2006906. 30278047 10.1371/journal.pbio.2006906 PMC6168121

[3] Katsanis SH. Pedigrees and perpetrators: uses of DNA and genealogy in forensic investigations. Annu Rev Genom Hum Genet. 2020;21:535–564. 10.1146/annurev-genom-111819-084213 32289230

[4] Kennett D. Using genetic genealogy databases in missing persons cases and to develop suspect leads in violent crimes. Forensic Sci Int. 2019;301:107–117. 31153988 10.1016/j.forsciint.2019.05.016

[5] Kling D, Phillips C, Kennett D, et al. Investigative genetic genealogy: current methods, knowledge and practice. Forensic Sci Int: Gen. 2021;52:102474. 10.1016/j.fsigen.2021.102474 33592389

[6] Phillips C. The Golden State Killer investigation and the nascent field of forensic genealogy. Forensic Sci Int: Gen. 2018;36:186–188. 10.1016/j.fsigen.2018.07.010 30041097

[7] Thomson J, Clayton T, Cleary J, et al. An empirical investigation into the effectiveness of genetic genealogy to identify individuals in the UK. Forensic Sci Int: Gen. 2020;46:102263. 10.1016/j.fsigen.2020.102263 32114291

[8] Formici G. From “familial searching” to “forensic genetic genealogy”: new frontiers—and challenges—of DNA analysis in criminal investigations. BioLaw J-Rivista di BioDiritto. 2021;21:S305–328.

[9] Ram N, Murphy EE, Suter SM. Regulating forensic genetic genealogy. Science. 2021;373:1444–1446. 34554804 10.1126/science.abj5724

[10] Scudder N, McNevin D, Kelty SF, et al. Policy and regulatory implications of the new frontier of forensic genomics: direct-to-consumer genetic data and genealogy records. Curr Issues Crim Justice. 2019;31:194–216.

[11] Skeva S, Larmuseau MH, Shabani M. Review of policies of companies and databases regarding access to customers’ genealogy data for law enforcement purposes. Personal Med. 2020;17:141–153. 10.2217/pme-2019-0100 32125932

[12] Wickenheiser RA. Forensic genealogy, bioethics and the Golden State Killer case. Forensic Sci Int: Synerg. 2019;1:114–125. 32411963 10.1016/j.fsisyn.2019.07.003 PMC7219171

[13] Michael AR, Blatt SH. The triad approach for human identification: the role of biological anthropologists in collaborative forensic genetic genealogy efforts. Forensic Genom. 2021;1:60–71.

[14] Blatt SH, Michael AR, Mittelman D. Leveraging anthropology, forensic genomics, and genealogy to restore identity to human remains. Intl Symp Hum Identif (ISHI). 2020; May 2020. Available from: https://promega.foleon.com/theishireport/may-2020/holistic-approaches-to-case-work-leveraging-anthropology-forensic-genomics-and-genealogy-to-restore-identity-to-human-remains/

[15] Ubelaker D, Bones SH. A forensic detective’s casebook. New York (NY): Harper Collins; 1992.

[16] Sill EP. Clark County Coroner’s Report; 1979.

[17] Kerley ER. The microscopic determination of age in human bone. Am J Phys Anthropol. 1965;23:149–163. 5826273 10.1002/ajpa.1330230215

[18] Kerley ER, Ubelaker DH. Revisions in the microscopic method of estimating age at death in human cortical bone. Am J Phys Anthropol. 1978;49:545–546. 216268 10.1002/ajpa.1330490414

[19] Ubelaker DH. Case Report (sent to FBI). September 20, 1979.

[20] Ousley SD, Jantz RL. Fordisc 3.1. computerized forensic discriminant functions. (Program Version 3.1.315); 2005.

[21] Spradley MK, Jantz RL. Sex estimation in forensic anthropology: skull versus postcranial elements. J Forensic Sci. 2011;56:289–296. 21210801 10.1111/j.1556-4029.2010.01635.x

[22] Stewart TD. Essentials of forensic anthropology, especially as developed in the United States. Spingfield (IL): Charles C. Thomas, 1979.

[23] McKern TW, Stewart TD. Skeletal age changes in young American males. The United States of America army quartermaster research and development command, technical report EP-45. Natick (MA); 1957.

[24] Todd TW. Age changes in the pubic bone. 1. The male white pubis. Am J Phys Anthropol. 1920;3:285–339.

[25] Dudar JC, Pfeiffer S, Saunders SR. Evaluation of morphological and histological adult skeletal age-at-death estimation techniques using ribs. J Forensic Sci. 1993;38:677–685. 8515218

[26] Lovejoy O, Meindl RS, Pryzbeck TR, et al. Chronological metamorphosis of the auricular surface of the ilium: a new method for the determination of adult skeletal age at death. Am J Phys Anthropol. 1985;68:15–28. 4061599 10.1002/ajpa.1330680103

[27] Işcan MY, Loth SR, Wright RK. Metamorphosis at the sternal rib end: a new method to estimate age at death in white males. Am J Phys Anthropol. 1984;65:147–156. 6507605 10.1002/ajpa.1330650206

[28] Yoder C, Ubelaker DH, Powell JF. Examination of variation in sternal rib end morphology relevant to age assessment. J Forensic Sci. 2001;46:223–227. 11305422

[29] Schneider CA, Rasband WS, Eliceiri KW. NIH image to ImageJ: 25 years of image analysis. Nat Methods. 2012;9:671–675. 22930834 10.1038/nmeth.2089 PMC5554542

[30] Schindelin J, Arganda-Carreras I, Frise E, et al. Fiji: an open-source platform for biological-image analysis. Nat Methods. 2012;9:676–682. 22743772 10.1038/nmeth.2019 PMC3855844

[31] Burr DB, Ruff CB, Thompson DD. Patterns of skeletal histologic change through time: comparison of an archaic Native American population with modern population. Anat Rec. 1990;226:307–313. 2327603 10.1002/ar.1092260306

[32] Miszkiewicz JJ, Mahoney P. Ancient human bone microstructure in medieval England: comparisons between two socio-economic classes. Anat Rec (Hoboken). 2016;299:42–59. 26480030 10.1002/ar.23285

[33] Skedros JG, Mason MW, Bloebaum RD. Differences in osteonal micromorphology between tensile and compressive cortices of a bending skeletal system: indications of potential strain-specific differences in bone microstructure. Anat Rec. 1994;239:405–413. 7978364 10.1002/ar.1092390407

[34] Stout SD, Crowder C. Bone remodeling, histomorphology, and histomorphometry. In: Crowder C. Stout SD, editors. Bone histology: an anthropological perspective. Boca Raton (FL): CRC Press; 2011. p. 1–21.

[35] Reichs KJ. Postmortem dismemberment: recovery, analysis, and interpretation. In: Reichs KJ, editor. Forensic osteology: advances in the identification of human remains. 2nd ed. Springfield (IL): Charles C Thomas; 1998. p. 353–388.

[36] Rajs J, Lundström M, Broberg M, et al. Criminal mutilation of the human body in Sweden—a thirty-year medico-legal and forensic psychiatric study. J Forensic Sci. 1998;43:563–580. 9608692

[37] Rainwater CW. Three modes of dismemberment: disarticulation around the joints, transection of bone via chopping, and transection of bone via sawing. In: Passalacqua NV, Rainwater CW, editors. Skeletal trauma analysis: case studies in context. Hoboken (NJ): John Wiley & Sons, Ltd.; 2015. p. 222–245.

[38] Symes SA, Chapman EN, Rainwater CW. Knife and saw toolmark analysis in bone: a manual designed for the examination of criminal mutilation and dismemberment. Doc No 232864. US Department of Justice; 2010. Available from: https://www.ncjrs.gov/pdffiles1/nij/grants/232864.pdf

[39] Erlich Y, Shor T, Pe’er I, et al. Identity inference of genomic data using long-range familial searches. Science. 2018;362:690–694. 30309907 10.1126/science.aau4832 PMC7549546

[40] DNA Painter 2021. Available from: https://dnapainter.com

[41] Drusini AG, Carrieri A, Frigo AC. Patterns of femoral bone remodeling: comparison of the Tongariki Native Eastern islanders with European populations. Open Anthropol J. 2010;3:96–106.

[42] Robling AG, Stout SD. Histomorphology, geometry, and mechanical loading in past populations. In: Agarwal SC, Stout SD, editors. Bone loss and osteoporosis: an anthropological perspective. New York (NY): Kluwer Academic/Plenum Publishers; 2003. p. 189–206.

[43] Gocha TP, Agnew AM. Spatial variation in osteon population density at the human femoral midshaft: histomorphometric adaptations to habitual load environment. J Anat. 2016;228:733–745. 26708961 10.1111/joa.12433 PMC4831343

[44] Pfeiffer S, Crowder C, Harrington L, et al. Secondary osteon and Haversian canal dimensions as behavioral indicators. Am J Phys Anthropol. 2006;131:460–468. 16685724 10.1002/ajpa.20454

[45] Frost HM. Secondary osteon populations: an algorithm for determining mean bone tissue age. Am J Phys Anthropol. 1987;30:221–238.

[46] Bartelink EJ, Chesson LA. Recent applications of isotopic analysis to forensic anthropology. Forensic Sci Res. 2018;1:29–44. 10.1080/20961790.2018.1549527 PMC6427615 30915415

[47] Christensen AM, Hefner JT, Smith MA, et al. Forensic fractography of bone: a new approach to skeletal trauma analysis. Forensic Anthropol. 2018;1:32–51.

[48] Isa MI, Fenton TW, DeLand TS, et al. Assessing impact direction in 3-point bending of human femora: incomplete butterfly fractures and fracture surfaces. J Forensic Sci. 2018;63:38–46. 28436033 10.1111/1556-4029.13521

[49] Tucker BK, Hutchinson DL, Gilliland MF. Microscopic characteristics of hacking trauma. J Forensic Sci. 2001;46:234–240. 11305424

[50] Konopka T, Strona M, Bolechała F, et al. Corpse dismemberment in the material collected by the Department of Forensic Medicine, Cracow, Poland. Leg Med (Tokyo). 2007;9:1–13. 17157050 10.1016/j.legalmed.2006.08.008

[51] Seidel AC, Fulginiti LC. The first cut is the deepest: looking for patterns in cases of human dismemberment. In: Martin DL, Anderson CP, editors. Bioarchaeological and forensic perspectives on violence: how violent death is interpreted from skeletal remains. Cambridge (UK): Cambridge University Press; 2014. p. 63–82.

[52] Kahana T, Aleman I, Botella MC. Postmortem dismemberment in two Mediterranean countries. J Forensic Indentif. 2010;60:557–572.

[53] Adams BJ, Rainwater CW, Yim A-D, et al. A retrospective study of intentional body dismemberment in New York City: 1996–2017. J Forensic Sci. 2019;64:1012–1016. 30707769 10.1111/1556-4029.14012

[54] Hawkins M. A short history of machine knitting; 2015. Available from: https://knittinghistory.co.uk/resources/a-short-history-of-machine-knitting/

[55] Over the State. The Idaho Republican, May 19, 1916. Available from: https://www.newspapers.com/clip/41483929/walter-cairins-aka-charles-smith-aka/

[56] Kidston M, Fifer B. Wanted!: wanted posters of the Old West. Helena (MT), Farcountry Press; 2003.

[57] Walter cairns escapes from jail. Caribou county sun, May 25, 1916. Available from: https://www.newspapers.com/paper/caribou-county-sun/889/?id=889&title=caribou-county-sun

[58] Ubelaker DH, Shamlou A, Kunkle A. Contributions of forensic anthropology to positive scientific identification: a critical review. Forensic Sci Res. 2019;4:45–50. 30915416 10.1080/20961790.2018.1523704 PMC6427489

[59] Bytheway JA, Connor M, Dabbs GR, et al. The ethics and best practices of human decomposition facilities in the United States. Forensic Sci Pol Management: Int J. 2015;6:59–68.

[60] Damann FE. Human decomposition ecology at the University of Tennessee Anthropology Research Facility [dissertation]. Knoxville (TN): University of Tennessee; 2010.

[61] Stoddard B, editor. Trails of the Silver Sage. Idaho Falls (ID): Clark County Historical Society; 2019.

[62] Scudder N. Privacy and the search for suspects using forensic genetic genealogy. Privacy Law Bull. August, 2020. Available from: https://www.lexisnexis.com.au/__data/assets/pdf_file/0003/404481/Privacy-and-the-search-for-suspects-using-forensic-genetic-genealogy-_-2020-175-PRIVLB-78_Attach2.pdf

[63] Samuel G, Kennett D. Problematizing consent: searching genetic genealogy databases for law enforcement purposes. New Gen Soc. 2021;40:284–304.

[64] McEwen J, Pino N, Raphael A, et al. Investigative genetic genealogy: ethical, legal, and social issues and directions for future research. Forensic Genom. 2021;1:91–98.

[65] Ubelaker DH. Radiocarbon analysis of human remains: a review of forensic applications. J Forensic Sci. 2014;59:1466–1472. 25041129 10.1111/1556-4029.12535

